# Special Issue “Mental Health Issues and Quality of Life in Older Individuals”

**DOI:** 10.3390/life11030221

**Published:** 2021-03-10

**Authors:** Omar Cauli, Rut Navarro-Martínez, Julio Fernández-Garrido

**Affiliations:** 1 Department of Nursing, University of Valencia, 46010 Valencia, Spain; Rut.Navarro@uv.es (R.N.-M.); Julio.Fernandez@uv.es (J.F.-G.); 2 Frailty and Cognitive Impairment Group (FROG), University of Valencia, 46010 Valencia, Spain; 3 Chair of Healthy, Active and Participatory Aging, Valencia City Council, University of Valencia, 46010 Valencia, Spain

There are numerous biological, psychological, and social factors that have a more or less prominent impact on the mental health of older adults. Apart from components derived from the normal processes of aging or the co-occurrence of various medical diseases, events such as the death of a loved one, retirement, or disability contribute significantly to a variety of mental or emotional problems at this stage of the life cycle. The most frequent problems affect the neurocognitive, affective, and sleep functions, which can lead to a reduction in quality of life. Major neurocognitive disorders reduce a patient’s overall performance and thus create demanding needs for dependency and a higher level of frailty. Affective disorders can be accentuated by a lack of family support and a marked decrease in social interactions, which can lead to significant isolation with resulting suicidal behaviour. The increased frequency of sleep disorders such as insomnia, daytime sleepiness, and specific disorders such as obstructive apnoea significantly can further alter the quality of life of this population. The complexity of these disorders needs the expertise of a multidisciplinary team to provide the best healthcare for these patients. 

The goal of this thematic issue was to shed new light on this exciting and insightful field of research from a multidisciplinary perspective. This issue of *Life*, entitled ‘‘Mental Health Issues and Quality of Life in Older Individuals”, reflects the interplay between geriatrics, nursing, and neurological and psychiatric sciences with other health sciences at the leading edge of this growing research field and explores new opportunities for improving the care or preventing adverse outcomes in several disorders or clinical situations. In this Special Issue, readership will find relevant research studies carried out by several health care professionals and researchers with extensive knowledge in the clinical setting that address new issues that are of particular importance to research and clinical practice. 

Buigues et al. [1] investigated the role of psycho-social factors in the efficacy of a cardiovascular prevention and rehabilitation programme in patients with coronary heart disease within the framework of the European-cluster randomised controlled trial EUROACTION study. The authors reported the importance of psycho-social factors; for example, less threatening illness perceptions are related to improved cardiovascular health behaviours and lower anxiety and depression levels, and the inclusion of family as support in patients’ long-lasting changes in behaviour. Fernández-Ruiz et al. [2] analysed the effectiveness of telehealth consultation for the re-evaluation of nutritional status and quality of life assessment in older people diagnosed with oropharyngeal dysphagia associated with active use of thickeners to prevent hospital admissions during the COVID-19 pandemic. The quality of life of these patients was observed to change according to the risk of malnutrition, which is associated with a decrease in some blood markers, such as albumin and total proteins’ concentration. 

Frias et al. [3] evaluated the factors associated with informal caregivers’ quality of life of individuals with dementia. Caregiver burden, psychological wellbeing, and negative aspects of caregiving on health all correlated with informal caregivers’ quality of life. The findings demonstrated that the course of the dementia illness and its severity with the presence of comorbid neuropsychiatric symptoms can negatively affect the informal caregiver’s role and produce a lower quality of life. Montoro Pazzini Watfe et al. [4] analysed the prevalence of disability and areas of life affected by disability among elders of the public primary health care in two big cities in Brazil. Both participation and mobility were the areas of life most affected by disability (ranging between 50–60% in this population). The high prevalence of disability and associated risk factors indicates that public primary health care is not meeting the needs of elders in primary care settings. 

Pérez-Ros and Martínez-Arnau [5] evaluated the psychometric properties of the self-reported EQ-5D (an instrument to quantify quality of life) in nursing home residents with cognitive impairment to analyse its validity based on scales included in the comprehensive geriatric assessment, showing that it represent a good tool for this purpose in such clinical settings. Pérez-Belmonte et al. [6] analysed major depressive disorder in older individuals from a representative Spanish sample. The results showed the presence of three different classes of major depressive disorder (e.g., psychosomatic, melancholic, and anhedonic (the most prevalent type)). This work represents a step forward in understanding the heterogeneity of major depressive disorder, facilitating the diagnosis and/or subsequent treatment of older adults. 

The review article by El Mlili et al. [7] summarized the current scientific evidence regarding the changes in the concentration of cortisol in hair cortisol and sleep disorders. Many previous studies have indicated that hair cortisol constitutes a valuable tool to further supplement existing data on salivary, plasma, or urinary cortisol concentrations in patients with sleep disorders.

## References

[1] Buigues C., Queralt A., De Velasco J.A., Salvador-Sanz A., Jennings C., Wood D., Trapero I. (2021). Psycho-Social Factors in Patients with Cardiovascular Disease Attending a Family-Centred Prevention and Rehabilitation Programme: EUROACTION Model in Spain. Life.

[2] Fernández-Ruiz V.E., Paredes-Ibáñez R., Armero-Barranco D., Sánchez-Romera J.F., Ferrer M. (2021). Analysis of Quality of Life and Nutritional Status in Elderly Patients with Dysphagia in Order to Prevent Hospital Admissions in a COVID-19 Pandemic. Life.

[3] Frias C.E., Cabrera E., Zabalegui A. (2020). Informal Caregivers’ Roles in Dementia: The Impact on Their Quality of Life. Life.

[4] Montoro Pazzini Watfe G., Fajersztajn L., Ribeiro E., Rossi Menezes P., Scazufca M. (2020). Prevalence of Older Adult Disability and Primary Health Care Responsiveness in Low-Income Communities. Life.

[5] Pérez-Ros P., Martínez-Arnau F.M. (2020). EQ-5D-3L for Assessing Quality of Life in Older Nursing Home Residents with Cognitive Impairment. Life.

[6] Pérez-Belmonte S., Galiana L., Sancho P., Oliver A., Tomás J.M. (2020). Subtypes of Depression: Latent Class Analysis in Spanish Old People with Depressive Symptoms. Life.

[7] El Mlili N., Ahabrach H., Cauli O. (2021). Hair Cortisol Concentration as a Biomarker of Sleep Quality and Related Disorders. Life.

